# Case Report: α-amanitin toxicosis leading to acute death in a puppy

**DOI:** 10.3389/fvets.2025.1542020

**Published:** 2025-06-18

**Authors:** Zachary Lake, Tasia Ludwik, Caylie Hake

**Affiliations:** Department of Veterinary Clinical Sciences, College of Veterinary Medicine, University of Minnesota, Saint Paul, MN, United States

**Keywords:** amanita, toxicity, canine, hypoglycemia, hepatic necrosis

## Abstract

A 12-week-old, male intact, Shetland Sheepdog presented with acute onset vomiting and diarrhea, rapidly progressing to stupor and hypoglycemic shock following ingestion of α-amanitin-containing mushrooms. Despite aggressive therapeutic interventions, the patient exhibited rapid systemic deterioration characterized by recurrent hypoglycemia, hypotension, and multi-organ failure, leading to cardiopulmonary arrest within 22 h of presentation. Definitive diagnosis was unable to be elucidated prior to death, leading to an untailored treatment plan. Post-mortem analysis confirmed extensive necrosis of the liver, kidneys, and brain. Presence of α-amanitin was confirmed in the hepatic tissue via post-mortem liquid chromatography–tandem mass spectrometry analysis. Serum collected at presentation was submitted post-mortem for an insulin level which was found to be discordantly elevated, which may demonstrate an alternative mechanism of hypoglycemia in this case. This case highlights the rapidly lethal potential of α-amanitin in pediatric patients and the non-classical case presentation. This report contributes to the limited veterinary literature on this toxin in pediatric patients and underscores the need for heightened awareness and rapid diagnosis and treatment in suspected cases.

## 1 Introduction

While overall prevalence varies based on season and geographic location, ingestion of the fruiting body of the *Amanita* genus of mushrooms often carries high morbidity and mortality in veterinary patients. According to the ASPCA Animal Poison Control Center, most reports of ingestion occur in the Northeast (40%) and West (23%) of the United States (1). These mushrooms most commonly appear in late summer and fall, and are often associated with oak, birch, and pine trees (2). The toxic elements of these mushrooms are cyclic peptides such as amatoxin, phallotoxin, or cycloamanide, with amatoxin being the primary element of interest due to its severe effects (3). Amatoxin is extremely toxic to veterinary species with the LD50 in dogs being as little as 0.1 mg/kg when administered intravenously (4). Ingestion of a single mushroom can be potentially fatal in dogs.

The clinical course of toxicity is well documented in humans and generally follows four phases characterized by a latency phase, a gastrointestinal phase, an apparent recovery phase, and a fulminant organ dysfunction phase (3). After ingestion, amatoxin exerts its first effects directly on the cells of the duodenum and ileum causing vomiting, cramping, and diarrhea approximately 6–8 h after ingestion (3). The toxin’s chemical stability prevents its breakdown by heat and acidity, facilitating ready intoxication via the gastrointestinal tract. The toxin is then absorbed into the bloodstream and taken up primarily by hepatocytes and later by other cells with high metabolic activity including the proximal convoluted tubule of the kidney, where it is a potent and specific inhibitor of RNA polymerase II decreasing transcription of mRNA leading to gradual cessation of protein synthesis and cell death (5). This gradual cessation leads to an apparent remission phase in which animals appear to recover after a few hours to days. The decreased protein synthesis continues and often progresses to fulminant hepatic necrosis and death within 2–3 days (2). In pediatric dogs, death may occur more rapidly, potentially within 24 h, and is generally associated with severe hypoglycemia (6, 7). Hypoglycemia is associated with hepatic glycogen depletion in fulminant hepatic failure (7), however direct stimulation of insulin release from the pancreatic islet cells has also been implicated as a potential mechanism (3, 8). To the author’s knowledge, no veterinary literature has reported changes in serum insulin levels in patients affected by α-amanitin.

The purpose of this case report is to outline the atypical clinical course of this toxin in a pediatric canine patient resulting in acute death, emphasize the importance of early diagnosis to develop a tailored treatment plan, and to provide the first supportive evidence in veterinary medicine that exposure may lead to hyperinsulinemia.

## 2 Case description

A 12-week-old male intact (MI) Shetland Sheepdog was presented to the University of Minnesota Veterinary Medical Center Emergency service (UMN VMC ER) for evaluation of acute onset vomiting and diarrhea. The evening prior to presentation, he was taken on a walk through the fairground campsite where they were staying, no abnormal behavior or foreign material ingestion was noted to occur. He ate dinner as normal and was placed in his kennel for the evening with no abnormalities noted. Seven hours later, he developed liquid diarrhea and vomiting. Three hours following initial gastrointestinal signs, the patient was noted to be unresponsive and was immediately transported to UMN VMC ER for evaluation.

At the time of presentation, the patient was laterally recumbent with a stuporous mentation. He exhibited sinus tachycardia (220 BPM), with tachypnea (60 BPM) and hyperthermia (104.6 F). He was 8–10% dehydrated with evidence of hypovolemic shock. He was noted to have malodorous green diarrhea containing small pieces of plastic and rubber.

Point-of-care (POC) lab work revealed severe hypoglycemia (21 mg/dL; Reference Interval (RI): 81-125 mg/dL), moderate hypokalemia (3.0 mmol/L; RI: 3.4–4.9 mmol/L), hemoconcentration (PCV 52%, TS 8.2 mg/dL), and a severe hyperlactatemic, titrational acidosis (pH 7.15; RI: 7.30–7.47, HCO3 9.7 mmol/L; RI: 17.8–27.2 mmol/L, BE −19 mmol/L; RI: −7 to 3, Lac 11.54 mmol/L; RI: 0.31–3.80 mmol/L, AG 19; RI: 8–17). Parvovirus SNAP test was negative. 1 mL/kg of 50% dextrose diluted 1:1 with 0.9% saline was administered intravenously, resulting in only a slight improvement in mentation. Lactated Ringer’s solution was administered intravenously as a bolus of 20 mL/kg, and the patient improved from stuporous to severely obtunded with an improvement in perfusion parameters. A complete blood count, serum chemistry, and coagulation panel were obtained and submitted 15 min after the initial POC lab work was performed and the first dextrose bolus had been administered during stabilization (Table 1). Due to ongoing evidence of hypovolemia based on perfusion parameters and a persistently abnormal mentation, 5 mL/kg of 7.5% hypertonic saline was administered resulting in no improvement in mentation. Point of care ultrasound (POCUS) was performed and revealed left ventricular end-systolic cavity obliteration and no peritoneal effusion. Discrete acoustic shadowing in the colon, along with the presence of foreign material in the diarrhea, raised concern for foreign body ingestion. An additional 20 mL/kg of Lactated Ringer’s solution was administered due to persistent hypovolemia. Ampicillin/sulbactam (Unasyn®) was administered at a dose of 30 mg/kg IV due to concern for occult septic peritonitis.

**Table 1:** Complete blood count, chemistry panel, and coagulation panel.

	Reference interval	Result
WBC (×1,000/μL)	3.88–14.57	4.36
SEG (×1,000/μL)	2.10–11.20	3.13
BAND (×1,000/μL)	0.00–0.13	0.24
LYMPH (×1,000/μL)	0.78–3.36	0.24
MONO (×1,000/μL)	0.00–1.20	0.32
EOS (×1,000/μL)	0.00–1.20	0.04
BASO (×1,000/μL)	0.00–0.13	0.00
HCT (%)	37.5–60.3	42.7
RETIC (×1,000/μL)	6–82	165
PLT (×1,000/μL)	129–395	356
BUN (mg/dL)	9–31	19
CREAT (mg/dL)	0.6–1.6	0.7
Ca (mg/dL)	9.3–11.5	11.6
Phos (mg/dL)	3.3–6.8	6.7
Mg (mg/dL)	1.7–2.4	2.1
Alb (g/dL)	2.7–3.7	2.9
Glob (g/dL)	1.7–3.5	2.0
Na (mmol/L)	145–153	149
Cl (mmol/L)	109–118	113
K (mmol/L)	3.6–5.3	3.1
HCO3 (mmol/L)	15.0–25.0	7.8
AG	15–28	31
T-bili (mg/dL)	0.0–0.3	0.5
ALP (U/L)	8–139	236
GGT (U/L)	0–6	7
ALT (U/L)	22–92	444
AST (U/L)	16–44	1,422
CK (U/L)	36–348	590
Glu (mg/dL)	75–117	134
Chol (mg/dL)	143–373	275
Amyl (U/L)	275–1,056	146
PT (s)	6.1–8.2	12.7
aPTT (s)	12.4–18.7	22.5
Fibrinogen (s)	98–232	336
D-Dimer (ng/mL)	No RI	2,415

Following initial fluid resuscitation and metabolic stabilization, severe abdominal pain was noted. Constant rate infusions (CRI) of fentanyl (4 mcg/kg/h) and lidocaine (25 mcg/kg/min) were initiated. Despite correction of hypovolemia and resolution of hypoglycemia, the patient’s mentation did not significantly improve and he remained obtunded despite normalized perfusion parameters. He was admitted to the Intensive Care Unit for ongoing therapy and monitoring. At the time of admission to ICU, primary differentials included gastrointestinal obstruction with secondary septic peritonitis and toxin ingestion (primarily xylitol).

A central venous jugular catheter was placed without complication. Abdominal radiographs were consistent with diffuse enteritis with no appreciable obstructive gas pattern or pneumoperitoneum. At this time, the clinical pathology profiles that had been submitted previously were made available (Table 1). Complete blood count revealed an absence of leukocytosis with bandemia and toxic change. A reticulocytosis was also noted. Serum chemistry which was drawn following the initial dextrose bolus administration revealed hypokalemia, hypobicarbonatemia, elevated anion gap, and mild hyperglycemia with a mixed, but primarily hepatocellular, hepatopathy with a discordant rise in AST. The coagulation profile revealed mild prolongations of PT and aPTT, moderate hyperfibrinogenemia, and elevated D-dimers. Notably at this time, aside from glucose, other markers of hepatic synthesis such as BUN, cholesterol, and albumin were normal. This decreased the likelihood that hypoglycemia was due solely to synthetic hepatic failure and further raised suspicion for septic consumption or xylitol toxicity.

Despite ongoing fluid support, the patient developed hypotension suspected secondary to ongoing losses and vasoplegia. An additional 10 mL/kg of Lactated Ringer’s and 5 mL/kg of Vetstarch were administered to assess for fluid responsiveness, resulting in resolution of hypotension. He was started on enrofloxacin 10 mg/kg IV for broad spectrum antimicrobial coverage and was continued on fentanyl, lidocaine, and intravenous fluids with potassium and 2.5% dextrose supplementation. Gastrointestinal support included maropitant 1 mg/kg IV and ondansetron 1 mg/kg IV. A Fresh Frozen Plasma transfusion (15 mL/kg IV over 5 h) was administered for suspected vasculitis and ongoing oncotic support. Monitoring and nursing care included telemetric ECG monitoring, continuous invasive blood pressure monitoring, recumbency care, and urinary catheterization for quantification of urine output.

Four hours from presentation, the patient developed recurrent, severe hypoglycemia (28 mg/dL; RI: 81–125 mg/dL) despite continuous dextrose supplementation. An additional dextrose bolus was administered, and continuous supplementation was increased to 5%. Recheck POCUS showed resolution of left ventricular end-systolic cavity obliteration and subjectively adequate contractility with no evidence of peritoneal effusion. The patient developed recurrent hypotension despite adequate volume status and was started on a norepinephrine CRI (0.1–0.3mcg/kg/min). N-acetylcysteine (140 mg/kg IV followed by 70 mg/kg IV q6) was added for hepatic support.

Six hours from initial presentation the patient developed a new-onset ionized hypercalcemia with a persistent hyperlactatemic, titrational acidosis and increased ScvO2 (Table 1), further increasing the concern for maldistribution and dysoxia. At this time, the patient was able to be weaned from norepinephrine and remained normoglycemic with supplementation. Antibiotic therapy in the form of Unasyn^®^ was continued as a CRI, and a low dose Vetstarch CRI was added for oncotic support due to continuous mucoid diarrhea leading to suspected low colloid osmotic pressure. Urine output (UOP) at that time revealed polyuria at 3 mL/kg/h. POCUS revealed a large, fluid filled stomach. With persistent abdominal pain and the patient’s altered mentation, a nasogastric tube was passed to relieve fluid distension and decrease risk of aspiration. A thick mucinous fluid was recovered. Abdominal discomfort appeared to be worsening as mentation improved through the evening, and ketamine was initiated for multimodal pain control.

Twelve hours from presentation, blood glucose was found to be trending toward hypoglycemia despite supplementation, and was increased to 7.5% via the central venous catheter. Hypotension recurred which was volume responsive. Polyuria was continuous with a UOP of 3 mL/kg/h. Mentation remained severely obtunded despite maintenance of normotension and normoglycemia.

Sixteen hours from initial presentation, blood pressure and blood glucose remained normal. Urine output decreased from polyuria to oliguria over the next 4 h despite apparent normovolemia and euhydration. Eighteen hours from presentation urine output had further decreased to 0.25 mL/kg/h with a USG of 1.024.

Twenty hours from presentation, the patient developed recurrent hypotension with a persistent tachycardia and anuria that was unresponsive to fluid challenge. Point of care lab work revealed a new azotemia (Creatinine 2.3 mg/dL, increased by 1.9 since presentation; RI: 0.8–1.5 mg/dL) and moderate hyperkalemia (6.3 mmol/L; RI: 3.4–4.9 mmol/L). Blood glucose was normal (121 mg/dL). Venous blood gas revealed progressive hyperlactatemic, titrational acidosis and decreased ScvO2 (Table 2). Cardiopulmonary arrest occurred 21 h from initial presentation and CPR was initiated. CPR was discontinued after 28 min due to owner decision. Remains were submitted for diagnostic necropsy.

**Table 2:** Point-of-care blood work values throughout hospitalization.

	Reference interval	Presentation	6 h	20 h
Na^+^ (mmol/L)	140–150	147	155	152
K^+^ (mmol/L)	3.4–4.9	3.0	3.6	6.3
Cl^−^ (mmol/L)	112–120	119	130	131
BUN (mg/dL)	8–35	17	14	26
Creat (mg/dL)	0.8–1.5	0.4	0.6	2.3
Glu (mg/dL)	81–125	21	103	121
iCa (mg/dL)	5.1–5.9	5.8	7.2	6.3
Anion gap	8–17	19	16	17
Hct (%)	38–60	52	38	30
pH	7.30–7.47	7.149	7.169	7.047
HCO_3_ (mmol/L)	17.8–27.2	9.7	12.3	8.8
BE (mmol/L)	-7-3	-19	−16	−22
ScvO2 (%)	56–90	66	79	35
Lactate (mmol/L)	0.31–3.8	11.54	7.73	8.90

### 2.1 Post-mortem examination

Necropsy revealed widespread necrosis in multiple organ systems. Hemoperitoneum was noted suspected to be secondary to cardiopulmonary resuscitation efforts as no peritoneal effusion was noted prior, although underlying progressive coagulopathy cannot be ruled out.

**Figure 1:**
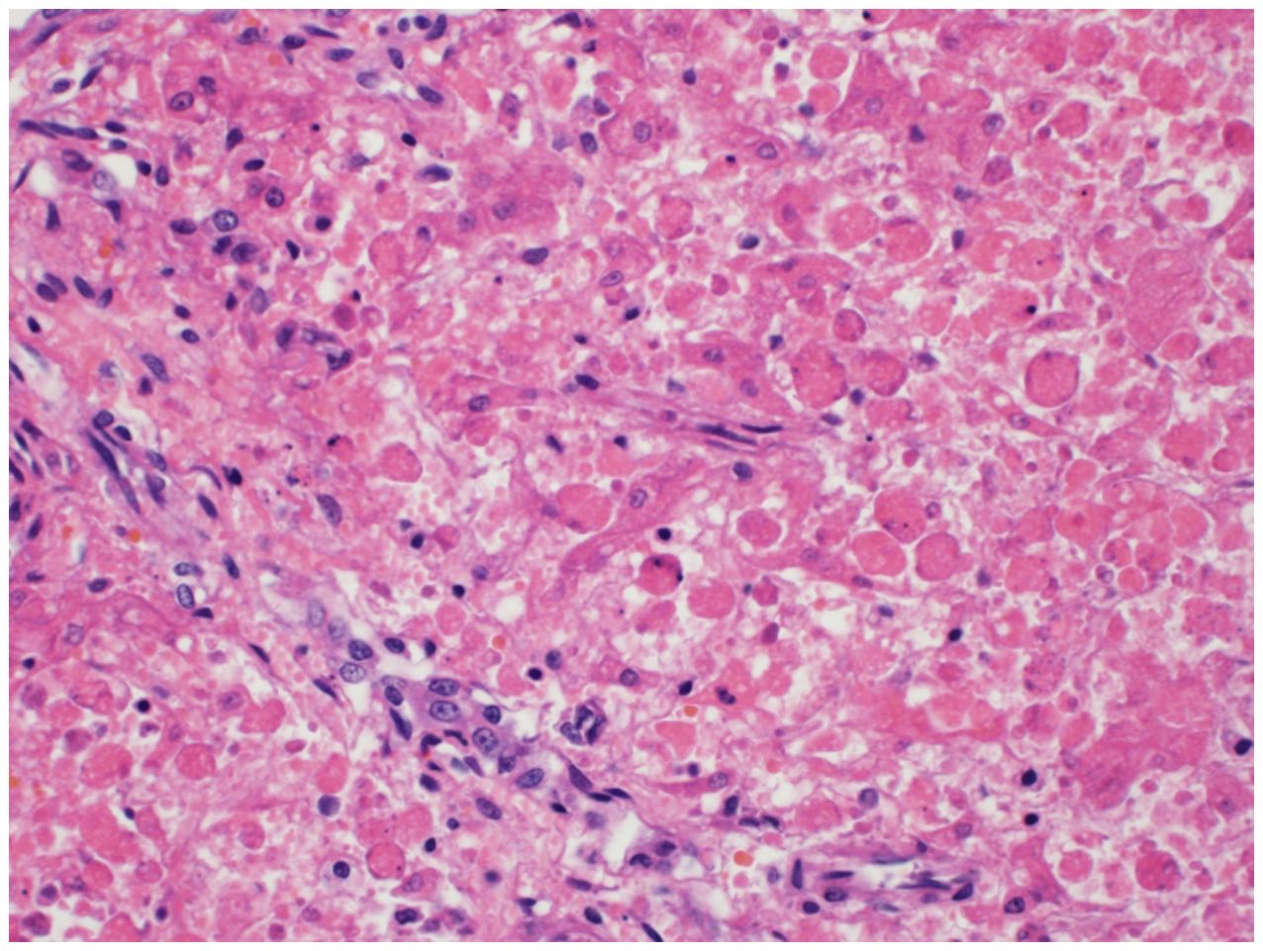
Hepatocellular necrosis. There is diffuse disruption of the hepatocyte cords. Individualized hepatocytes exhibit variable cell size, rounding of cytoplasmic margins, and cytoplasmic hypereosinophilia. Most of the hepatocytes lack a nucleus and others have karyorrhectic or pyknotic nuclear remnants.

**Figure 2:**
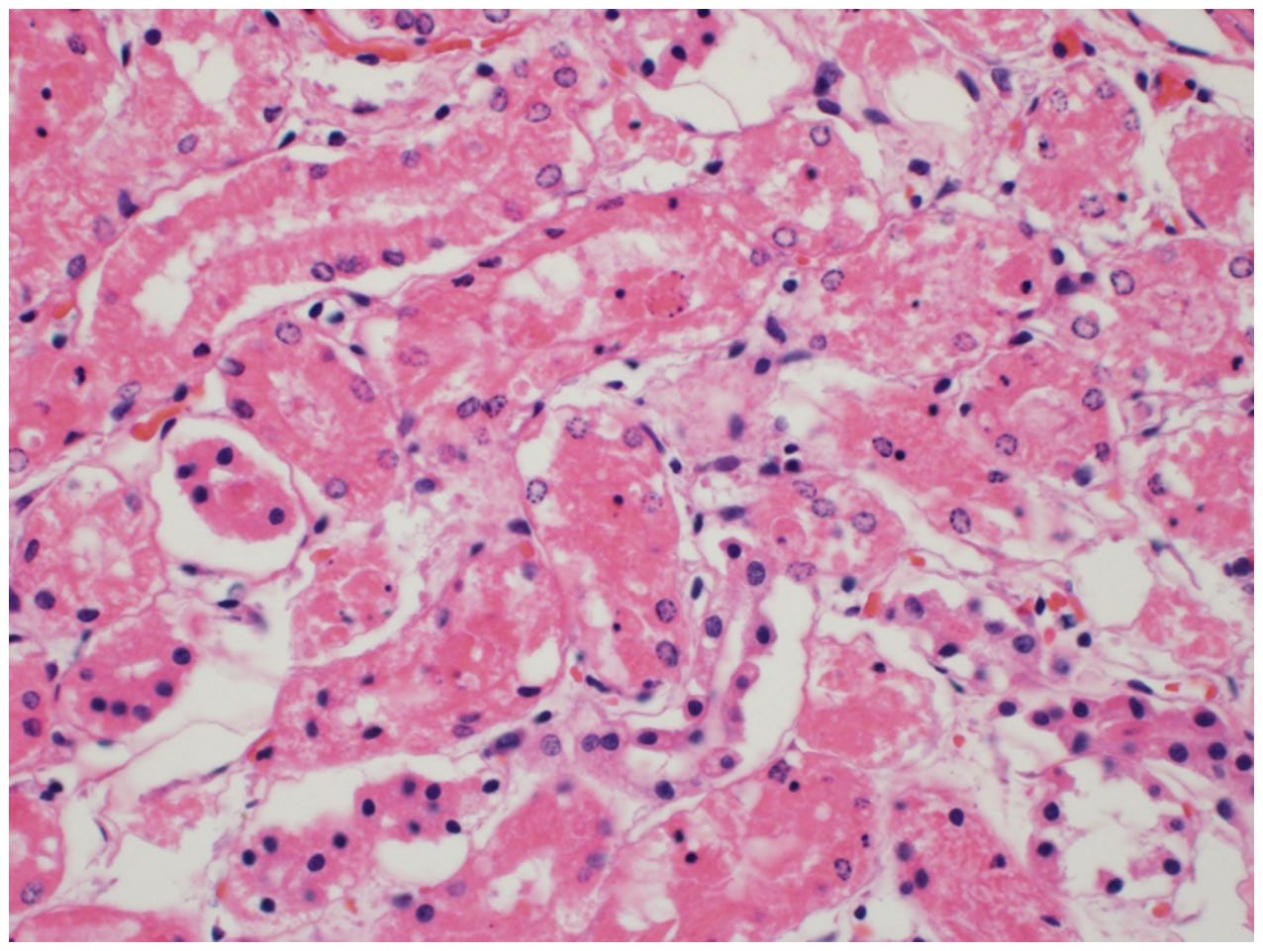
Renal cortical tubule epithelial necrosis. The cortical tubules are commonly affected by necrosis of the epithelial cells characterized by cytoplasmic vacuolation and/or hypereosinophilia, nuclear pyknosis, karyorrhexis, or karyolysis, and sloughing into the tubule lumina. Sloughed epithelial cells are admixed with amorphous, eosinophilic debris within the tubular lumina.

Histopathology of the liver revealed massive (diffuse), acute hepatocellular necrosis characterized by disruption of hepatocyte cords and centrilobular, mid-zonal, and periportal cellular coagulative necrosis along with grossly appreciated multifocal fissures suspected to be due to compressions (Figure 1). The renal cellular architecture was found to have varied morphology including vacuolation, swelling, shrinking, karyolysis, pyknosis, karyorrhexis, and tubular sloughing. Amorphous eosinophilic material was noted in a majority of cortical tubule lamina. Medullary tubules were also noted to contain granular, eosinophilic debris which often filled the entire lumen. This is consistent with moderate to marked cortical tubular epithelial necrosis (Figure 2).

**Figure 3:**
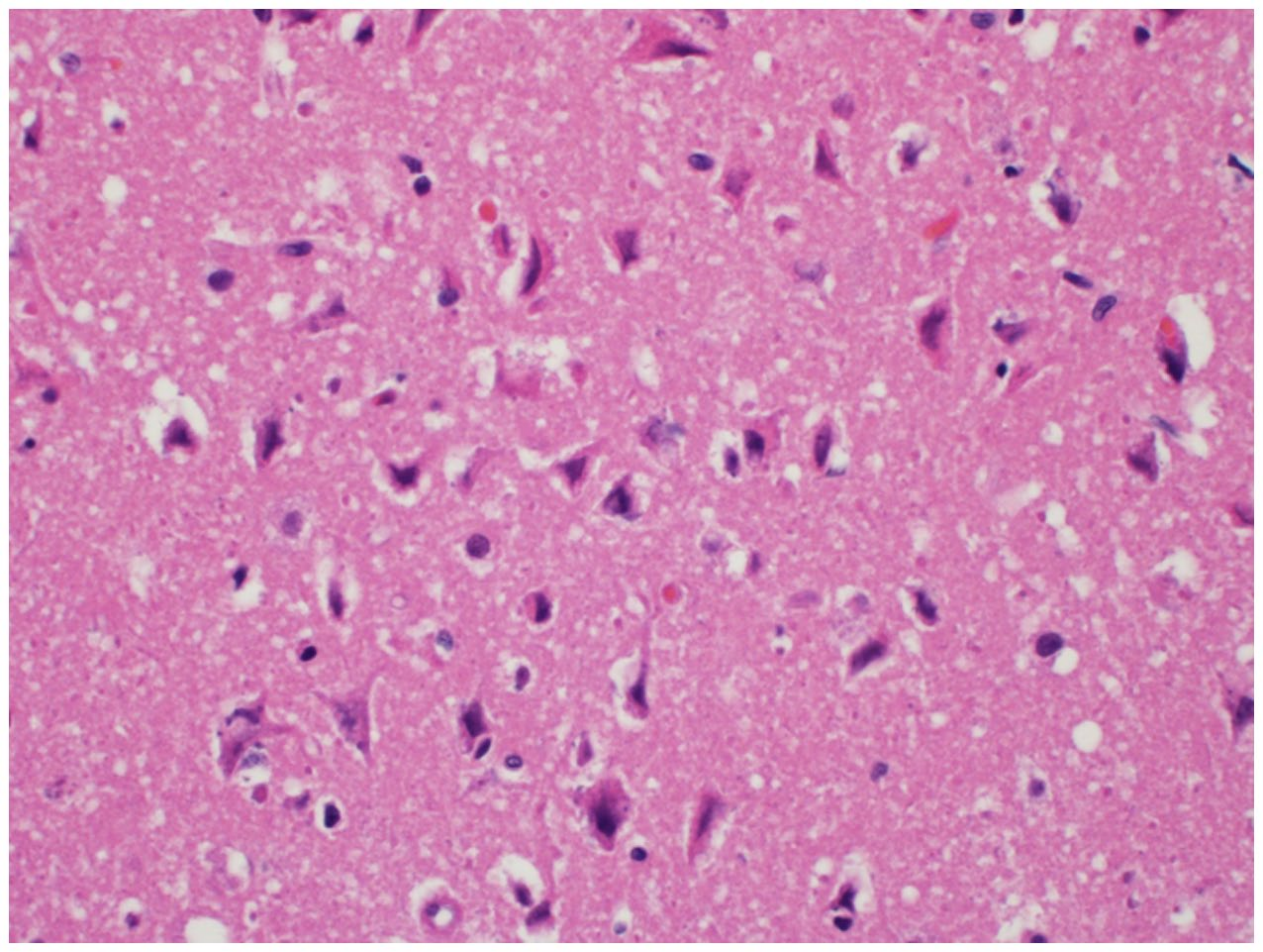
Neuronal necrosis. Neurons within the deep cerebrocortical laminae exhibit neuronal degeneration and necrosis characterized by shrunken, angular cell bodies with hypereosinophilic cytoplasm and karyolytic or pyknotic nuclei. The neurons and blood vessels are commonly surrounded by a clear space indicative of edema.

Histopathology of the brain showed 10–20% of the cerebral cortical neurons of the forebrain and thalamus were hypereosinophilic and somewhat angular with indistinct or absent nuclei. Areas surrounding the neurons and small vessels were also noted to be clear. These findings are consistent with widespread, moderate, acute laminar neuronal necrosis with associated cerebrocortical edema (Figure 3).

Finally, acute, mild, widespread alveolar edema was noted in the lung along with bacteria consistent with *Helicobacter* sp. within the gastric mucosa.

### 2.2 α-amanitin detection

After performing retrospective case evaluation and literature review, the differential of α-amanitin toxicity was prioritized. Additionally, the owners later reported identifying decomposing mushrooms of unknown type near the campsite. At the request of the primary clinician, hepatic tissue was submitted to the University of California-Davis Animal Health and Food Safety Laboratory System for qualitative detection of α-amanitin. The tissue was processed and samples extracted for analysis by liquid chromatography–tandem mass spectrometry (LC–MS) for α-amanitin. Trace concentrations were detected in the hepatic tissue, which confirms exposure to amanitin-containing mushrooms in this case.

### 2.3 Insulin levels

Patient serum that was collected at the time of CBC/Chemistry submission was submitted to the Michigan State University Veterinary Diagnostic Laboratory for analysis of insulin levels. This showed that a moderate to severe hyperinsulinemia (72.5μIU/mL; RI: 8.1–31.9μIU/mL) was present despite only mild hyperglycemia (130 mg/dL; RI: 81–118 mg/dL) with an elevated Insulin: Glucose (I: G) ratio (0.56; RI: 0.11–0.33).

## 3 Discussion

This case report outlines the presentation, response to therapy, diagnostic results, and clinical outcome of a puppy with confirmed α-amanitin exposure. This toxin accounts for the majority of confirmed cases of mushroom poisonings in animals (1), however there remains a relative deficit in literature involving these cases. Although α-amanitin toxicosis classically follows the four-stage progression, this case highlights the variability in clinical course that can be encountered, especially in juvenile animals. This toxin can cause mortality in any age of dog, but as has been seen in other cases involving young dogs and is exemplified in this case, the progression can be accelerated and lead to mortality within 24 h (6, 7, 9–11).

This case underscores the potential benefits of early screening for α-amanita toxicosis in patients presenting with severe gastrointestinal signs and hypoglycemia. Accurate and early diagnosis was made difficult in this case specifically due to the rapid case progression and inability to rule out other differentials. Severe hypoglycemia with only moderately elevated liver enzymes, led to high suspicion of a consumptive etiology, such as septic peritonitis or xylitol toxicity. The suspicion of septic peritonitis was also initially prioritized due to the presence of multiple signs of severe systemic inflammation including low normal neutrophils with bandemia which may be indicative of consumption, hyperthermia, tachycardia, and tachypnea. This also could have explained the coagulation changes seen consistent with early disseminated intravascular coagulation such as an elevated fibrinogen indicative of ongoing coagulation and elevated D-dimers representing the degradation of already formed microthrombi. This potential for unregulated systemic inflammation caused by a septic nidus led to a less targeted treatment plan which included antimicrobial therapy, hepatic support, colloidal support, glycemic support, plasma administration and other supportive care. Recently a targeted regimen, the Modified Santa Cruz Protocol, adapted from human medicine, has shown promise in successfully treating α-amanitin toxicosis in dogs. This protocol resulted in all 5 dogs treated surviving to discharge (12). Classically, this intoxication has had a mortality rate approaching 80% (13). This protocol involves treatments that may be considered invasive (i.e., cholecystocentesis) or not ideal for a hypoglycemic puppy (i.e., strict NPO), making accurate diagnosis paramount. Typically, a presumptive diagnosis is made based on witnessed or suspected ingestion, presence of the classical pathophysiologic stages and is later confirmed by detection of α-amanitin via liquid chromatography-mass spectrometry (LC–MS) in serum, urine, gastric contents, liver, kidney, or provided mushroom sample (7). This test is highly sensitive but has a turnaround time of 10–14 days. Alternatively, a POC lateral flow immunoassay (AMATOTOXtest^®^) has been developed which has a higher detection level compared to LC–MS but is relatively cheap and can be performed in minutes on samples of mushroom or urine and has been shown to provide a more rapid diagnosis (14, 15). This case may further demonstrate the utility of this bedside test, as an antemortem diagnosis may have led to altered treatment plan, following the Modified Santa Cruz Protocol, and case outcome.

Conventionally, hypoglycemia in cases of α-amanitin toxicosis is thought to be secondary to hepatic insufficiency and glycogen depletion, although in this case hepatic synthetic parameters were normal at the time of hypoglycemia. Some evidence has demonstrated that α-amanitin may have effects on the β-cells of the pancreas, stimulating insulin release (16). Serum insulin levels in this case were found to be elevated comparable to levels found in patients with insulin secreting tumors (8). This may be suggestive of α-amanitin induced insulin secretion in this case, however the only serum available for analysis was collected at the time of initial chemistry submission after dextrose had been administered and serum glucose was mildly elevated. The I: G ratio was also elevated to twice the value of the upper reference limit which may provide additional support that the insulin concentration was discordantly elevated. To the author’s knowledge, the only other report of an I: G ratio being performed on a patient with known exposure was not consistent with hyperinsulinemia, making relevance of this potential process difficult to determine (13). Further research into this aspect of toxicity is required to elucidate the clinical implications of this occurrence, but it is important that clinicians do not rule out this toxin as a differential of hypoglycemia even if hepatic synthetic parameters are not yet affected.

Any suspicion for ingestion of *Amanita* mushrooms should be taken seriously as multiple organ failure can rapidly occur with the liver, kidneys, and brain all showing histopathological necrosis in this case. Hepatic necrosis with this toxicity has been visualized before (7) and anuria has been reported (9), but to the author’s knowledge there has not been histopathologic confirmation of renal tubular necrosis (Figure 2) or laminar neuronal necrosis (Figure 3) in the veterinary literature. With the importance of maintaining adequate glomerular filtration rate to eliminate toxic elements and the possibility of oliguria/anuria, urinary catheterization for careful urinary output monitoring may be beneficial. Urinary output monitoring was performed in this case and did identify oliguria, but the patient arrested prior to additional therapies aside from ensuring adequate volume status.

Mentation change has been reported with the progression to fulminant hepatic necrosis. This has been attributed mostly to hepatic encephalopathy, intracranial hypertension, or cerebral edema seen with acute liver failure (13, 17). Acute neuronal necrosis has been reported with hypoglycemia in a dog with an insulinoma (18), but has not yet been reported in a case of α-amanitin toxicosis. Laminar neuronal necrosis has also been diagnosed following ischemia, fluid overload, carbon monoxide exposure, and infarction in dogs (19–26). This finding may explain why the mentation did not improve markedly throughout hospitalization despite cardiovascular stabilization and normoglycemia. Worsening hepatic function and hepatic encephalopathy may also have been contributing, but repeated hepatic parameters were not available. The possibility of return to function following laminar neuronal necrosis is not known, but this likely affects overall prognosis as a previous case series reported that most of the patients that did not survive hospitalization were euthanized due to grave prognosis caused by deteriorating mental status (13).

This case report documents the fatal outcome of a pediatric Shetland Sheepdog with α-amanitin toxicosis and provides the first documentation of hyperinsulinemia in a veterinary patient with this condition. The rapid progression in this case led to limitations in data with no sequential hepatic values and lack of specialized abdominal imaging. The case underscores the complexity of diagnosing and managing α-amanitin toxicity, particularly in young animals, and suggests that hyperinsulinemia may play a role in the pathophysiology of hypoglycemia with amanita toxicosis. Further studies are needed to understand the clinical prevalence of this mechanism and to adjust treatment protocols. Ingestion of α-amanitin containing mushrooms should remain a top differential for acute onset hypoglycemia with gastrointestinal signs despite unwitnessed ingestion and low geographical prevalence, and should be treated rapidly and aggressively.

## Data Availability

The original contributions presented in the study are included in the article/supplementary material, further inquiries can be directed to the corresponding author.
